# Implications of evolutionary engineering for growth and recombinant protein production in methanol-based growth media in the yeast *Pichia pastoris*

**DOI:** 10.1186/s12934-017-0661-5

**Published:** 2017-03-17

**Authors:** Josef W. Moser, Roland Prielhofer, Samuel M. Gerner, Alexandra B. Graf, Iain B. H. Wilson, Diethard Mattanovich, Martin Dragosits

**Affiliations:** 1 Department of Chemistry, University of Natural Resources and Life Sciences, Muthgasse 11, 1190 Vienna, Austria; 2 Austrian Centre of Industrial Biotechnology (ACIB), 1190 Vienna, Austria; 3 Department of Biotechnology, University of Natural Resources and Life Sciences, Vienna, Austria; 4 University of Applied Sciences FH-Campus Wien, Bioengineering, Vienna, Austria

**Keywords:** *Pichia pastoris*, Experimental evolution, Methanol, Recombinant protein

## Abstract

**Background:**

*Pichia pastoris* is a widely used eukaryotic expression host for recombinant protein production. Adaptive laboratory evolution (ALE) has been applied in a wide range of studies in order to improve strains for biotechnological purposes. In this context, the impact of long-term carbon source adaptation in *P. pastoris* has not been addressed so far. Thus, we performed a pilot experiment in order to analyze the applicability and potential benefits of ALE towards improved growth and recombinant protein production in *P. pastoris*.

**Results:**

Adaptation towards growth on methanol was performed in replicate cultures in rich and minimal growth medium for 250 generations. Increased growth rates on these growth media were observed at the population and single clone level. Evolved populations showed various degrees of growth advantages and trade-offs in non-evolutionary growth conditions. Genome resequencing revealed a wide variety of potential genetic targets associated with improved growth performance on methanol-based growth media. Alcohol oxidase represented a mutational hotspot since four out of seven evolved *P. pastoris* clones harbored mutations in this gene, resulting in decreased Aox activity, despite increased growth rates. Selected clones displayed strain-dependent variations for AOX-promoter based recombinant protein expression yield. One particularly interesting clone showed increased product titers ranging from a 2.5-fold increase in shake flask batch culture to a 1.8-fold increase during fed batch cultivation.

**Conclusions:**

Our data indicate a complex correlation of carbon source, growth context and recombinant protein production. While similar experiments have already shown their potential in other biotechnological areas where microbes were evolutionary engineered for improved stress resistance and growth, the current dataset encourages the analysis of the potential of ALE for improved protein production in *P. pastoris* on a broader scale.

**Electronic supplementary material:**

The online version of this article (doi:10.1186/s12934-017-0661-5) contains supplementary material, which is available to authorized users.

## Background

In the field of microbial biotechnology, the methylotrophic yeast *Pichia pastoris* (*Komagataella phaffii* [1]) is a commonly used host organism for recombinant protein production, in small scale lab applications as well as larger scale protein production. Several factors, including its ability to grow on methanol, the availability of constitutive as well as inducible promoter systems, its minimal growth requirements, high biomass yields and eukaryotic-type post-translational modifications led to the establishment of this yeast as a heterologous host [2]. More recently, whole-cell glyco-engineering has achieved humanized N-glycosylation patterns in this yeast [3, 4]. Furthermore, in the last decade several studies on the systems-level led to an improved understanding of *P. pastoris* physiology and the correlations of recombinant protein production and process-relevant environmental factors [5–7]. Thus, classical approaches, such as co-chaperone overexpression in order to increase production efficiency in bacteria and yeasts [8–10], can be complemented by systems-wide analysis in order to identify novel targets for strain and process engineering.

Although glucose- and glycerol based expression systems are also widely applied for *P. pastoris* [11], methanol-induced expression of recombinant proteins mediated by strongly inducible promoters such as the alcohol oxidase 1 (Aox1) gene promoter [12] can be considered as one of the core features of *P. pastoris*. Despite the toxicity and flammability of methanol, leading to the development of novel methanol-free expression systems in *P. pastoris* [13], recent approaches led to the identification of new potential methanol-inducible promoters and expression strategies for this expression system [14]. Consequently, methanol utilization and the Aox expression system have been studied in detail in recent years. The *P. pastoris* methanol utilization (Mut) phenotypes are commonly known as methanol utilization Mut^+^ (fast growth), Mut^s^ (slow growth) and Mut^−^ (no growth) and depend on the presence of one or two functional copies of the alcohol oxidase gene (*AOX1* and *AOX2*, respectively). Furthermore, the implications of the Mut^+^ and Mut^s^ phenotypes in recombinant production have been investigated but led to indistinct results in terms of recombinant protein productivity [15, 16]. The effect of co-overexpression of methanol-pathway genes was analyzed [15] and an *AOX* promoter mutant library was tested for protein production [17]. Recent studies also led to a better understanding of the methanol utilization process in general; e.g. methanol utilization is predominantly regulated on the transcriptional rather than on the translational level in *P. pastoris* [18], whereas other data show that methanol metabolic processes are confined to peroxisomes [19]. Additionally, transcriptional regulators involved in the expression of Mut proteins have been identified, clarifying how they work in concert to promote important steps such as Mut protein expression and peroxisome proliferation [20–22].

The investigation of evolutionary processes on a molecular level has become an intriguing topic in recent years. Thus, adaptive laboratory evolution (ALE) experiments with microbial cells led to important insights into evolutionary processes including the rate and progression of adaptation [23], pleiotropic effects [24], growth trade-offs and benefits in non-evolutionary growth conditions [25] among others. Harnessing the power of systems-level analysis and affordable genome sequencing technologies, it became also clear that such studies have significant potential for biotechnology, e.g. in the area of metabolic engineering and white biotechnology. Thus, the implications of ALE for biotechnology have been subject of recent reviews [26, 27].

To date it is not clear to which extent ALE experiments with *P. pastoris* can lead to improved growth on methanol and provide data that lead to a better understanding of host cell physiology and new strategies for host cell engineering. Furthermore, the impact of potentially increased growth rates due to long-term environmental adaptation on recombinant protein production is not understood. To address this question, we performed a serial passaging ALE experiment with consecutive transfers in batch cultures to maintain replicate *P. pastoris* populations in rich- and minimal medium with methanol as carbon source for 250 generations. Whereas there are several different methods for experimental evolution, serial passaging and chemostat selection are two highly preferred methods [27, 28]. Each of these methods has its benefits and trade-offs. Although microbial populations experience fluctuating conditions in serial batch cultures, the gradual reduction of lag phase and long exponential growth phase can easily lead to the selection for improved growth rates [28]. On the other hand, chemostat cultivation allows growth under strictly nutrient-limiting conditions and tight control of other environmental parameters, such as pH [28]. Nevertheless, this nutrient-limiting growth does not necessarily lead to improved growth rates in non-limiting conditions and due to the activation of nutrient scavenging mechanisms it has been reported to result in overall decreased stress resistance of microbial cells [29, 30]. Thus, in order to select for improved growth rates during growth on methanol in two different environmental settings, serial dilution was the method of choice. After ALE we performed growth tests with evolved populations on a broader scale and selected individual clones from both growth environments for whole genome sequencing in order to identify mutations associated with the observed growth phenotypes. Finally, selected clones were tested for recombinant protein production in small-scale and larger scale fed batch cultures.

## Methods

### *P. pastoris* and *Escherichia coli* strains

*Pichia pastoris* X-33 was used as model strain in the current study. Cloning steps were performed using *E. coli* JM109 cells.

### Long-term cultivation on methanol as carbon source

*Pichia pastoris* cells were adapted to growth on either YPM medium (YP medium pH 7.4, 1% methanol) or BMM (buffered minimal medium [12], 1% methanol). For each condition, four populations were cultivated in 24-deep well plates (10 mL total reservoir volume, 2 mL culture volume) at 28 °C, 200 rpm on an orbital shaker (Thermo MaxQ 4000, orbit diameter 1.9 cm). For both growth environments, populations were transferred to a fresh deep-well plate in 24-h intervals. Daily dilutions of 1:20 and 1:10 were used for YPM and BMM medium, respectively. Due to a lower growth rate and higher fluctuations of cell densities, the lower dilution rate of 1:10 was necessary for BMM in order to maintain a stable serial transfer. The OD_600_ of each culture was determined on a daily basis (Tecan M200 plate reader) in order to calculate daily generations and the cumulative number of cell divisions (CCD). Long-term cultivation was performed for a total of approximately 250 generations. Cultures were checked for contamination by microscopy and plating samples on YPD-agar plates (plates were incubated at 28 °C for 2 days) every 50 generations. The calculation of CCD was essentially performed as described previously by Lee and co-workers [31], assuming 5 × 10^7^ cells mL^−1^ per OD_600_ unit [12].

### Growth tests in deep-well plates

For growth profiling in various growth media, yeast populations were grown in glass tubes (2 mL culture volume) in the respective growth medium at 28 °C, 200 rpm over night. On the next day, 2 mL cultures in deep-well plates were inoculated at a starting OD_600_ between 0.05 and 0.1 and growth was monitored for 8–12 h in order to calculate growth rates (µ_max_) and after 24 h (48 h for BMM cultures) for final OD_600_ (Tecan M200 plate reader) values.

### Genome re-sequencing and analysis

Genomic DNA of selected clones was isolated from o/n cultures grown in YPD (28 °C, 200 rpm) using the Masterpure Yeast DNA isolation kit (Epicentre). Illumina MiSeq paired-end sequencing with 300 bp read length was performed according to standard procedures using chemistry v3 at Eurofins Genomics (Eurofins Genomics NGS laboratory, Ebersberg, Germany). Cutadapt [32] was used for adapter-removal and quality filtering of the reads. For the genome assembly of the ancestral strain, Meraculous was applied [33]. The final assembly of the ancestral strain consisted of 48 scaffolds with a total of 9,310,711 bp and a N50 score of 910,678. Out of 4,048,141 quality trimmed input reads with an average read length of 260,48 bp, 3,581,438 (88.47%) were used in the assembly. The mean coverage amounted to 97.6.

The assembly scaffolds were further mapped to the CBS 7435 reference strain [34] in order to obtain an ordered assignment to chromosomes. CONTIGuator [35] was used for this purpose. This resulted in four large sequences containing 44 joined scaffolds and 3 scaffolds with 2506, 1439, and 1035 bp length, which could not be assigned to the reference.

Augustus [36] was used for gene prediction. Annotation was performed by matching predicted genes against the CBS 7435 reference strain using blast. Variant-calling was performed using kSNP3 [37] and GATK [38]; Magnolya [39] and cn.mops [40] were applied in order to test for potential CNVs in the evolved strains. Co-assembly for Magnolya was performed with velvet [41]. Alignment files were manually reviewed using the Integrative Genomics Viewer [42]. Potential mutations identified in the evolved clones were verified by subsequent Sanger-sequencing (primer list in Additional file 1: Table S1).

### Alcohol oxidase activity

2 mL of yeast cultures were grown in deep-well plates in YP or buffered minimal medium containing 1% glucose at 28 °C, 200 rpm over night. Cultures on YPM and BMM were started at an OD_600_ of 0.2 and samples for AOX activity were taken after 6 h during exponential growth. The collected cell pellets were washed with 1× PBS and stored at −20 °C until further use. For AOX activity assays, pellets were resuspended in ice-cold 1× PBS and treated by two freeze–thaw cycles. The suspension was used directly for AOX activity assays. Alcohol oxidase assays were performed as follows: 40 µL of cell suspension with an OD_600_ of 2.5 were combined with 10 µL HRP solution (2 mg mL^−1^ in HQ-H_2_O), 50 µL 1% methanol and 200 µL ABTS [2,2′-azino-bis-(3-ethylbenzothiazoline-6-sulfonic acid), Sigma Aldrich, dissolved in 100 mM KH_2_PO_4_, pH 7.5]. Assays were incubated at 28 °C for 45–90 min and absorbance was read at 405 nm.

### Cloning and overexpression of PAS_chr2-1_0445

For the overexpression of the potential GAL4-like protein a DNA Hifi Assembly kit was used (New England Biolabs). The open reading frame was amplified from *P. pastoris* X-33 genomic DNA using Q5 Polymerase (New England Biolabs) and primers listed in Additional file 1: Table S1. The PCR-product was cloned into a pGAPzB vector backbone. *E. coli* competent cells were transformed by heat shock transformation. The insert sequence of positive clones was verified by Sanger sequencing. *P. pastoris* X-33 competent cells were used for the transformation with linearized versions of pGAPzB and pGAPzB-PAS_chr2-1_0455. Growth tests of four random clones for each construct were performed as described above.

### Recombinant gene expression

Selected clones were used for the expression of rHSA (recombinant human serum albumin, [43]) and recombinant *Drosophila* hexosaminidase (rFDL) [44]. Electro-competent *P. pastoris* cells from the various strains were prepared as described in literature [12] and used for transformation with a linearized pPICzαA_rFDL, harboring a codon-optimized FDL coding sequence or a linearized pPM2dZ30_pAOX_HSA plasmid for rHSA expression. Both genes were expressed using the AOX promoter.

High biomass batch cultures: Cultures were grown in YPD at 28 °C, 200 rpm for 24 h and used to inoculate 2 mL M2 (1% glucose [45]) minimal medium supplemented with slow-release glucose feed beads (Kuhner, Switzerland) at an OD_600_ of 2.0. These cultures were grown for further 22 h at 28 °C, 200 rpm. Afterwards cells were harvested and taken up in 1 mL M2 medium without carbon source. The suspension was used to inoculate M2 medium containing 0.5% methanol at an OD_600_ of approximately 4.0. Cultures were grown at 28 °C, 200 rpm for 48 h. Cultures were supplemented with additional 1% methanol at 6, 24 and 36 h.

Low biomass batch cultures: Cultures were grown in 2 mL YPD or BMD medium at 28 °C, 200 rpm over night. On the following day, 2 mL of YPM or BMM medium were inoculated at a starting OD_600_ of 0.2. Cultures were grown at 28 °C, 200 rpm for a total of 48 h. Feeding with 1% methanol occurred in 12 h intervals.

### Fed batch cultivations

rHSA-expressing *P. pastoris* clones were used for fed batch cultivations in parallel 1.0 L bioreactors (DASGIP, Germany). The clones used were selected based on similar rHSA protein levels achieved in batch cultivation (X-33 wt, Y250 3a, M250 1a and M250 3b, respectively). 100 mL YP-medium, 2% glycerol, starter cultures were grown in shake flasks at 28 °C, 200 rpm for 48 h. Cells were harvested by centrifugation, resuspended in sterile 1× PBS and used to inoculate 400 mL bioreactor batch cultures at an OD_600_ = 1. After the initial batch phase with a target biomass yield of 20 g YDM L^−1^, a glycerol fed-batch with culture-dependent feed rates of 2.2–5.0 g h^−1^ was performed for a target biomass yield of 40 g YDM L^−1^. Cultures were pulsed with methanol fed batch medium with a total of 0.5% methanol before methanol feeds were started. For the methanol feed, feed rates of 0.5, 1.0, 1.5 and 2.0 g h^−1^ were applied. The feed was stop between constant feed phases and cultures were pulsed several times with methanol fed batch medium (MeOH concentrations ranging from 0.75 to 1.5%, Additional file 1: Figure S1). For all cultivations, temperature was set to 28 °C during batch and glycerol fed batch. Starting with the methanol feed phase the temperature was set to 25 °C. DO was controlled at 20%, pH was set to 5.85 and controlled by the addition of 25% ammonia. 5% (w/w) antifoam solution (Glanapon 2000, Bussetti, Austria) was added on demand to prevent excessive foaming. Samples were taken at regular intervals. Biomass was determined in triplicate, by drying culture aliquots in pre-weighed tubes to constant weight.

Batch medium composition was essentially as described previously [46, 47] and contained (L^−1^): 28.1 g H_3_PO_4_, 0.6 g CaSO_4_·2H_2_O, 9.5 g K_2_SO_4_, 7.8 g MgSO_4_·7H_2_O, 2.6 g KOH, 40.0 g glycerol, 4.6 g PTM_0_ trace salts stock solution and 2.0 g biotin solution (0.2 g L^−1^). Glycerol fed batch medium consisted of (L^−1^): 724 g glycerol (86%), 10 g PTM_0_ and 1.7 g biotin solution (0.2 g L^−1^). Methanol fed batch medium consisted of (L^−1^): 988 g methanol, 12 g PTM_0_ and 2 g biotin solution (0.2 g L^−1^). The PTM_0_ trace salts stock solution contained (L^−1^) 6.0 g CuSO_4_·5H_2_O, 0.08 g NaI, 3.0 g MnSO_4_·H_2_O, 0.2 g Na_2_MoO_4_·2H_2_O, 0.02 g H_3_BO_3_, 0.5 g CoCl_2_, 20.0 g ZnCl_2_, 65.0 g FeSO_4_·7H_2_O and 5.0 mL H_2_SO_4_ (95–98%).

### rHSA and rFDL quantification

HSA was quantified using a HSA quantitation set (Bethyl laboratories). ELISA plates were coated with coating antibody (1:100) at 4 °C over night. After washing plates were blocked with 1% BSA at room temperature on a rotary shaker for 30 min. After washing, HSA standards and samples were applied to the plates and incubated at room temperature for 1 h. Plates were washed and the detection antibody (HRP-conjugate, 1:30,000) was added. After 1 h, ELISA plates were washed and TMB substrate was added. The detection reaction was stopped by the addition of 2 M H_2_SO_4_ and absorbance was measured at 450 nm. For fed batch cultures, rHSA was quantified using a Caliper Labchip-DS microfluidic instrument (Perkin Elmer) with BSA as standard protein.

rFDL was quantified by determining enzymatic activity in culture supernatants as described previously [44]. In short, 2 µL of appropriately diluted culture supernatant were combined with 38 µL 5 mM *p*-Nitrophenyl-β-d-*N*-Acetylglucosaminide (Sigma Aldrich) in McIlvaine buffer pH 4.0 and incubated at 30 °C for 1 h. 200 µL stop solution (0.4 M glycine pH 10.4) were added and absorbance was read at 405 nm.

## Results

### Long-term adaptation towards growth on methanol

Long-term adaptation was performed in YPM (rich) and BMM (minimal growth conditions) medium in order to analyze the implications of environmental specialization to different growth contexts on growth and recombinant protein production. *P. pastoris* populations were cultivated on YPM and BMM medium with a transfer to fresh growth medium in 24-h intervals for 58 and 75 days respectively, yielding a total of approximately 250 generations (Additional file 1: Figure S2). Previous studies showed that improved phenotypes could be successfully selected after 100 generations [27]. On average, the four parallel populations in complex (YPM) medium achieved 4.3 generations per day, whereas the replicate populations on BMM achieved 3.4 generations per day. The final cumulative number of cell divisions (CCD) ranged from 10^9.96^ to 10^10.02^ for YPM cultures, whereas the populations adapted to BMM minimal medium underwent a total of 10^9.63^–10^9.69^ cumulative cell divisions (Additional file 1: Table S2). Henceforth, populations evolved on YPM medium will be denoted Y250 1-4 and populations adapted to BMM medium M250 1-4, respectively.

In order to analyze the impact of the ALE experiment, all populations were tested in terms of growth rate (Table 1; Additional file 1: Table S3) and biomass yield (OD_600_, Table 2) in comparison with the ancestral strain. Three out of four Y250 populations showed significant growth rate improvements on YPM medium and all M250 populations showed significant improvements on BMM. Surprisingly, all YPM-adapted populations also showed significantly increased growth rates on BMM, but only the second M250 population (M250 2) showed a significant increase of growth rate on YPM. Similarly, we observe significantly increased or decreased growth rates in non-evolutionary growth conditions, including different growth media with glucose or glycerol as carbon source and additional NaCl-induced salt stress (Table 1). Interestingly, most populations also showed increased growth rates on YPD and, with the exception of populations Y250 1 and 4, a trend towards decreased growth rates on BMD. The latter was more pronounced in the M250 populations. Regarding the biomass yield, as determined by the measurement of OD_600_ values of the cultures after 24 h of growth, mostly minor effects were observed. The largest effect was observed for M250 populations which, in addition to the increased growth rate on BMM, also showed significantly increased optical density of, on average, 177% as compared with the ancestral strain. Furthermore, Y250 populations showed significantly reduced biomass yields on the non-evolutionary BMDN growth medium, yielding only 74% of the ancestral OD_600_. For the remaining conditions tested, only minor population-specific differences were observed (Table 2).

**Table 1:** Growth rates µ [h^−1^] of *P. pastoris* populations in different growth conditions

	YPM	BMM	YPD	YPDN	BMD	BMDN	YPG	BMG
X-33	0.209 ± 0.001	0.089 ± 0.009	0.308 ± 0.002	0.281 ± 0.007	0.312 ± 0.005	0.264 ± 0.007	0.321 ± 0.008	0.269 ± 0.004
X-33 Y250 1	*0.227* *±* *0.002*	*0.108* *±* *0.001*	*0.340* *±* *0.004*	0.278 ± 0.005	0.316 ± 0.007	0.269 ± 0.015	*0.353* *±* *0.006*	*0.250* *±* *0.003*
X-33 Y250 2	0.212 ± 0.006	*0.123* *±* *0.004*	*0.372* *±* *0.002*	0.282 ± 0.005	0.297 ± 0.008	*0.205* *±* *0.005*	*0.355* *±* *0.007*	0.265 ± 0.004
X-33 Y250 3	*0.217* *±* *0.005*	*0.142* *±* *0.001*	*0.359* *±* *0.005*	0.279 ± 0.02	0.302 ± 0.005	*0.316* *±* *0.014*	0.321 ± 0.004	*0.285* *±* *0.001*
X-33 Y250 4	*0.216* *±* *0.003*	*0.136* *±* *0.005*	*0.364* *±* *0.004*	0.291 ± 0.005	*0.348* *±* *0.011*	*0.356* *±* *0.005*	*0.354* *±* *0.004*	0.260 ± 0.002
X-33 M250 1	*0.192* *±* *0.007*	*0.129* *±* *0.002*	*0.334* *±* *0.002*	*0.183* *±* *0.003*	0.286 ± 0.015	0.246 ± 0.017	0.327 ± 0.002	0.257 ± 0.007
X-33 M250 2	*0.219* *±* *0.003*	*0.127* *±* *0.001*	*0.328* *±* *0.002*	0.260 ± 0.032	*0.258* *±* *0.009*	0.240 ± 0.019	*0.363* *±* *0.002*	*0.310* *±* *0.003*
X-33 M250 3	0.199 ± 0.005	*0.125* *±* *0.007*	*0.368* *±* *0.008*	0.264 ± 0.006	*0.259* *±* *0.010*	*0.200* *±* *0.013*	*0.350* *±* *0.004*	*0.302* *±* *0.011*
X-33 M250 4	0.214 ± 0.001	*0.126* *±* *0.005*	0.314 ± 0.03	0.270 ± 0.009	*0.256* *±* *0.004*	*0.215* *±* *0.005*	*0.278* *±* *0.013*	*0.289* *±* *0.008*

Growth tests were performed in 24-deep well plates as described in the “Methods” section. *X-33* ancestral strain, *X-33 Y250a-d* populations evolved on YPM medium, *X-33 M250a-d* populations evolved on BMM medium, *YPM* YP medium 1% MeOH, *BMM* buffered minimal medium 1% MeOH, *YPD* YP medium 2% glucose, *YPDN* YPD 500 mM NaCl, *BMD* buffered minimal medium 2% glucose, *BMDN* BMD 250 mM NaCl, *YPG* YP medium 2% glycerol, *BMG* buffered minimal medium 2% glycerol; values represent averages ± standard error (*n* = 4). Growth rates in italics differ significantly from X-33 ancestral growth rates (*p* < 0.05, Additional file 1: Table S3)

**Table 2:** The final OD_600_ of ancestral and evolved populations

	YPM	BMM	YPD	YPDN	BMD	BMDN	YPG	BMG
X-33 wt	2.65 ± 0.09	0.83 ± 0.01	6.46 ± 0.13	4.56 ± 0.22	4.07 ± 0.15	3.09 ± 0.26	6.05 ± 0.16	7.98 ± 0.32
X-33 Y250 1	2.67 ± 0.07	1.26 ± 0.02	7.24 ± 0.13	4.34 ± 0.15	4.20 ± 0.02	2.11 ± 0.09	4.23 ± 0.10	8.77 ± 0.22
X-33 Y250 2	2.94 ± 0.07	1.05 ± 0.02	7.27 ± 0.10	4.80 ± 0.07	4.78 ± 0.10	2.55 ± 0.09	4.01 ± 0.20	8.02 ± 0.17
X-33 Y250 3	2.96 ± 0.02	0.81 ± 0.01	7.69 ± 0.04	4.35 ± 0.04	4.33 ± 0.10	2.35 ± 0.11	4.87 ± 0.13	6.84 ± 0.09
X-33 Y250 4	3.13 ± 0.04	1.01 ± 0.02	7.60 ± 0.15	4.44 ± 0.05	4.31 ± 0.10	2.18 ± 0.08	4.74 ± 0.05	7 47 ± 0.15
X-33 M250 1	2.63 ± 0.0.3	1.42 ± 0.01	7.08 ± 0.20	4.57 ± 0.08	3.70 ± 0.22	4.04 ± 0.02	5.52 ± 0.16	7 26 ± 0.26
X-33 M250 2	2.59 ± 0.08	1.43 ± 0.02	6.86 ± 0.12	4 52 ± 0.04	3.53 ± 0.09	3 18 ± 0.15	5.96 ± 0.13	7.14 ± 0.22
X-33 M250 3	3.33 ± 0.05	1.62 ± 0.01	6.79 ± 0.14	4 59 ± 0.06	4.28 ± 0.12	3.52 ± 0.15	6.10 ± 0.17	6.08 ± 0.07
X-33 M250 4	2.61 ± 0.05	1.43 ± 0.04	7.25 ± 0.05	4 27 ± 0.05	4.28 ± 0.12	3.36 ± 0.17	5.19 ± 0.17	6.23 ± 0.20

Growth tests performed in 24-deep well plates as described in the “Methods” section. *X-33* ancestral strain, *X-33 Y250a-d* populations evolved on YPM medium, *X-33 M250a-d* populations evolved on BMM medium, *YPM* YP medium 1% MeOH, *BMM* buffered minimal medium 1% MeOH, *YPD* YP medium 2% glucose, *YPDN* YPD 500 mM NaCl, *BMD* buffered minimal medium 2% glucose, *BMDN* BMD 250 mM NaCl, *YPG* YP medium 2% glycerol, *BMG* buffered minimal medium 2% glycerol; Measurements were performed after 24 h of cultivation (BMM after 48 h). Values represent averages ± standard error (*n* = 4)

### Single clone growth characteristics

**Fig. 1:**
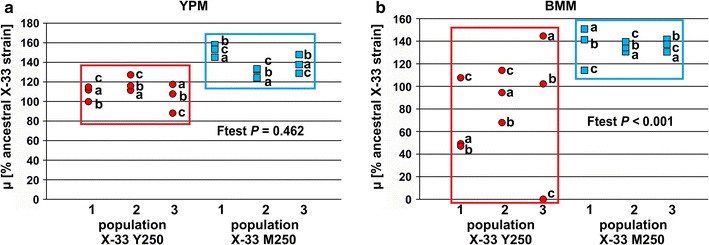
Single clone growth rates in deep-well cultures. Three single clones from YPM (*red circles*) and BMM-evolved (*blue squares*) population 1–3 were randomly selected and growth rates were compared to the ancestral *P. pastoris* strain on YPM (**a**) and BMM (**b**). % growth rate relative to the ancestral strain is shown. Number of replicates per single clone, *n* = 2

From three of the evolved populations three single clones were randomly selected and tested for growth rate on various growth media. As can be seen in Fig. 1a, b, most of these clones showed increased growth rate under growth conditions that were used for adaptation. Seven out of nine clones evolved on YPM medium showed increased growth rates on this growth medium. In contrast to the results for the population samples, five out of nine single clones showed decreased growth rates as compared with the ancestral strain on BMM medium (Fig. 1b). Unlike YPM-evolved clones, BMM-evolved clones showed higher growth rates on both media (140 and 135% as compared to the ancestral strain in contrast to 110 and 81% for the YPM-evolved clones) and significantly lower variance on YPM medium (Fig. 1a, b); thus, the growth rate of the single clones does not reflect the effects observed on the population level. Furthermore, growth of single clones was also tested on YPD growth medium with glucose as carbon source. Independent of growth medium type used for adaptation, evolved clones showed a decreased growth rate on YPD with, on average, 80% ancestral growth rate (Additional file 1: Figure S3).

### Genomic mutations in evolved *P. pastoris* clones

To identify potential mutations underlying the observed growth phenotypes after long-term adaptation, Illumina MiSeq whole genome sequencing was applied. Based on the single clone characterization results described in the previous section, the single clone with the highest growth rate in adaptive conditions from populations Y250 1-3 and M250 1-3 was selected (Y250 1c, 2c, 3a and M250 1a, 2c, 3b, respectively). Additionally, the ancestral strain and clone Y250 3c (Fig. 1b) were selected for resequencing. Clone Y250 3c was included since this clone showed the highest growth trade-off (almost no detectable growth) on BMM medium.

Illumina MiSeq paired-end 300 bp reads were mapped to the de-novo assembled ancestral strain which is covering approximately 91.1% of the CBS 7435 reference genome. An average 94-fold coverage was obtained for each sequenced clone (Additional file 2: Table S4). Mutations were found in each of the adapted clones. In total 17 mutations were identified, with the number for each individual clone ranging from two to four mutations (Table 3). 16 mutations represented point mutations with three mutations occurring in intergenic regions between open reading frames. The remainder of the mutations were identified within predicted genes and led to one premature stop codon and twelve amino acid conversions. In line with previous observations regarding mutation frequencies [26], G to A (31%) and C to T (12%) mutations were most frequent. We also found one insertion/deletion (Indel) mutation in one of the clones (Y250 3c). Although we applied two different methods for copy number variation (CNV) detection (see “Methods” sections for details), no potential CNVs were detected.

**Table 3:** Mutations in methanol-adapted *P. pastoris* clones

Strain	chr	Position	Type	Ref	Alt	Gene/locus	Effect
Y250 1c	chr. 1	1,477,448	SNP	G	A	Upstream of PAS_chr1-4_0035 (*SPC110*)	
	chr. 2	1,572,979	SNP	C	G	PAS_chr2-1_0445 (Zn_cluster)	G142 to R142
	chr. 4	237,622	SNP	G	A	PAS_chr4_0821 (*AOX1*)	W190 to stop
	chr. 4	1,574,048	SNP	G	A	PAS_chr4_0108 (*YCT1*) weak homology	A64 to T64
Y250 2c	chr. 2	1,573,676	SNP	G	T	PAS_chr2-1_0445 (Zn_cluster)	Upstream of gene
	chr. 3	627,999	SNP	C	A	PAS_chr3_0836 (*ECM22*)	W95 to C95
Y250 3a	chr. 3	313,093	SNP	G	T	PAS_chr3_1001 (*TUP1*)	C285 to F285
	chr. 3	628,000	SNP	C	A	PAS_chr3_0836 (*ECM22*)	W95 to L95
Y250 3c	chr. 2	2,100,561	SNP	C	T	PAS_chr2-1_0162 (*SLN1*)	R336 to K336
	chr. 4	238,206	indel	AAGACAAGCC	A	PAS_chr4_0821 (*AOX1*)	3 amino acid deletion after E385
M250 1a	chr. 2	1,060,279	SNP	G	A	PAS_chr2-1_0701 (*PKH3*)	G354 to D354
	chr. 3	384,863	SNP	C	T	PAS_chr3_0956 (*RRP45*)	G206 to D206
	chr. 3	575,412	SNP	C	T	Downstream of PAS_chr3_1229 (*SEC5*) and downstream of PAS_chr3_0322 (tRNA-Thr7)	–
M250 2c	chr. 1	1,737,822	SNP	A	C	PAS_chr1-4_0181 (*NMA1*)	Q118 to H118
	chr. 4	238,443	SNP	G	A	PAS_chr4_0821 (*AOX1*)	R464 to K464
M250 3b	chr. 3	1,260,818	SNP	G	A	PAS_chr3_0512 (*PAH1*)	C304 to Y304
	chr. 4	238,309	SNP	C	G	PAS_chr4_0821 (*AOX1*)	F419 to L419

Mutations were identified by WGS (Illumina mi-Seq). The type of mutation (Single nucleotide polymorphism—SNP or insertion/deletion—indel) as well as the DNA sequence of the ancestral strain (ref) and the sequence of the evolved clone (alt) is shown. Chromosomal position (chr) and nucleotide position on contigs is shown with respect to the *P. pastoris* CBS7435 reference sequence

### Convergent mutational targets in independently evolved *P. pastoris* clones

**Fig. 2:**
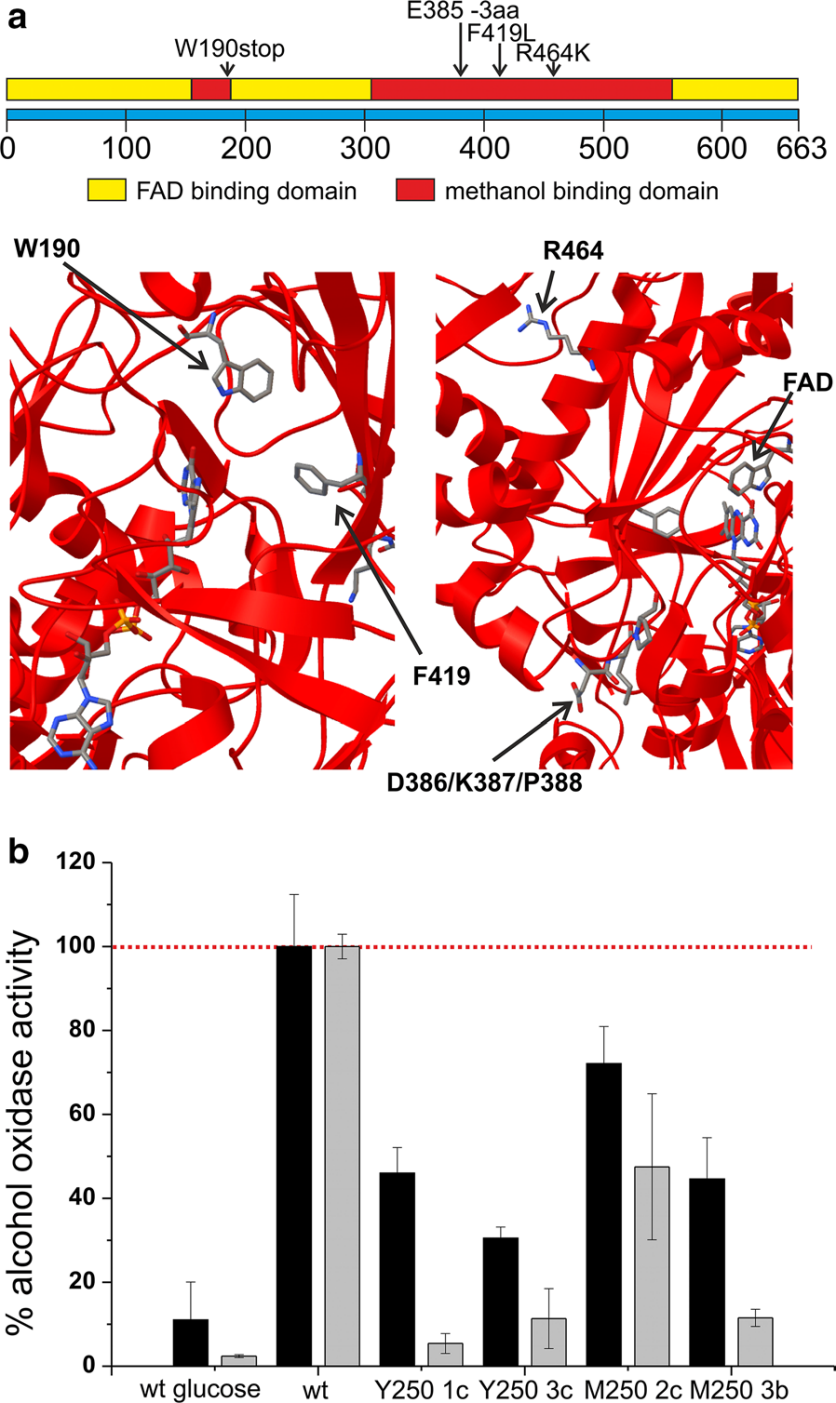
*AOX1* mutations and activity in several ancestral and evolved *P. pastoris* strains. **a** Aox1 protein domains according to the recently published crystal structure (PDB ID 5HSA) [49]. Amino acid positions mutated in the evolved strains are highlighted. **b** Alcohol oxidase activity in *P. pastoris* strains. Activity was determined as described in the “Methods” section. The enzymatic activity of wildtype *P. pastoris* X-33 on YPM and BMM growth medium was set to 100%. The activity of the wildtype strain on glucose and methanol is shown, as well as the activity of four evolved strains with mutations of the *AOX1* gene on methanol as carbon source. YPM (*black bars*), BMM (*grey bars*); Values represent averages of two biological and two technical replicates ±standard deviation

Adaptive evolution experiments often lead to a high degree of convergence in the selection of mutational targets. This convergence may be observed for distinct genetic loci or in terms of functional metabolic complexes [48]. In the current study, three genetic loci were affected in multiple independently evolved clones:

We found mutations in the alcohol oxidase 1 (*AOX1*) gene in four out of the seven evolved clones. Aox1 is the first enzyme in the methanol utilization pathway and catalyzes the conversion of methanol and oxygen into formaldehyde and hydrogen peroxide. Thus, it is not surprising that this key enzyme represents a potential selective target during the adaptation towards growth on methanol. Aox1 mutations were not limited to either rich or minimal growth conditions, but were found after long-term adaptation to both conditions (clones Y250 1c and 3c and M250 2c and 3b, respectively). Recently, the crystal structure of the *P. pastoris* Aox1 protein has been solved [49]. The enzyme monomer of the otherwise octameric protein structure has two main binding sites: the FAD cofactor binding site and the substrate binding site. The premature stop codon mutation in clone Y250 1c led to a heavily truncated protein, missing most of both sites. In clone Y250 3c, the deletion of amino acids 386–388 occurred in a loop region in proximity to the methanol binding site. The two amino acid conversions in the minimal medium evolved clones occurred in the methanol binding site (Fig. 2a). Since the truncated *AOX1* gene in clone Y250 1c most likely leads to a non-functional Aox1 enzyme, the alcohol oxidase activity was determined. Our data show that all four evolved clones with mutations in the *AOX1* gene exhibited lower alcohol oxidase activity as compared with the ancestral strain during exponential growth. This lower activity was irrespective of the growth medium, as all Y250 and M250 clones showed this reduced activity on both, YPM and BMM growth media (Fig. 2b). Aox activity was not abolished completely in mutated clones. As mentioned in the introduction, the *P. pastoris* genome encodes two *AOX* genes, *AOX1* and *AOX2*, respectively. Thus, the residual activity observed in the clones with mutated *AOX1* can be accounted for by *AOX2* expression (Fig. 2b).

A second convergent target was found in the open reading frame PAS_ch2-1_0445. Two evolved clones, both adapted to YPM growth medium, namely Y250 1c and 2c harbored a mutation linked to this predicted ORF. It encodes a putative Zn-cluster, GAL4-like transcription factor of unknown function. In order to analyze whether the encoded protein has any effect on the growth of *P. pastoris*, we overexpressed the gene in the X-33 wildtype background. Growth was assessed on glucose and methanol as carbon source in the context of both, YP and buffered minimal (BM) medium. Generally, the overexpression clones showed a tendency towards slightly decreased growth rates on both carbon sources on YP medium and a tendency towards increased growth rates on BMM medium (Additional file 1: Figure S4a, b). In terms of biomass yields, no differences were observed except from a significantly reduced biomass yield of the PAS_ch2-1_0445 overexpression cells on BMM medium (Additional file 1: Figure S4c, d).

Another GAL4-like yeast specific transcription factor with similarity to *S. cerevisiae ECM22*, involved in the regulation of sterol biosynthesis, was mutated in two independent clones. Similar to the mutations related to the open reading frame PAS_chr2-1_0445, these mutations occurred after adaptation towards YPM medium in the clones Y250 2c and 3a.

### Singular mutational targets

The remaining mutations were specific for the individual clones. The single mutations for YPM-evolved clones (Y250 1c, 3a and 3c) affected the genes *SPC110*, *PAS*-*chr4_0108*, *TUP1* and *SLN1*. SPC110 is a spindle pole body (SPB) component and PAS_chr4_0108, whose gene product belongs to the MFS general transporter family, shares weak homology with the *S. cerevisiae* gene for the Yct1 ER cysteine transporter. *TUP1* is an important transcriptional regulator involved in many physiological processes in yeast, including but not limited to stress response, mating as well as glucose sensing- and regulation [50]. Finally, clone Y250 3c had a SNP mutation in the transmembrane kinase *SLN1*, a transmembrane osmosensor critical for HOG pathway signaling [51].

Clone M250 1a harbored a SNP in PAS_chr2-1_0701, an ORF with similarity to the *S. cerevisiae PKH1/2/3* genes, whose gene products are regulators of protein serine/threonine kinase Pkc1. Furthermore this clone showed a SNP mutation in the *RRP45* gene, involved in cytoplasmic and nuclear RNA processing. The last mutation in this clone was intergenic between the genes for PAS-chr3_1229 (*SEC5*) and PAS-chr3_0322 (tRNA-Thr7) and also remains to be further analyzed in future studies. In addition to an *AOX1* mutation, the clone M250 2c differed from the ancestral strain in the *NMA1* gene, encoding a nicotinic acid mononucleotide adenylyltransferase involved in NAD synthesis. The evolved clone M250 3b had, in addition to an *AOX1* mutation, a nucleotide change in the *PAH1* locus, a phosphatidate phosphatase involved in the regulation of phospholipid biosynthesis.

### The effect of methanol adaptation on recombinant protein production

In order to analyze the impact of MeOH-adaptation on recombinant protein expression, we used the model proteins rHSA [43] and rFDL [44] for expression in the ancestral X-33 and selected evolved clones. Whereas rHSA is a protein where relatively high product titers can be achieved, rFDL is a difficult-to-produce protein in *P. pastoris* [44]. In a first experiment, rHSA was expressed using a standardized screening protocol in minimal growth medium, mimicking a high cell density limited fed batch culture. For the expression procedure, cultures were grown in a glucose batch culture followed by an 18 h glucose fed batch phase using glucose feed beads. At a relatively high biomass concentration (OD_600_ = 4), cultures were induced with methanol for recombinant protein expression and grown for additional 48 h with methanol feeding in regular intervals. As summarized in Table 4, clones evolved on YPM medium showed significantly lower rHSA titers as well as lower biomass yield than the ancestral wildtype host strain. Contrary, clones evolved on minimal medium, namely M250 1a and 3b, showed on average a 28 and 15% increase in rHSA titers as well a trend towards higher biomass yields, whereas the rHSA yield per biomass (OD_600_) was only higher for the clone M250 1a.

**Table 4:** Recombinant gene expression in ancestral and evolved *P. pastoris* X-33 strains

Strain	rHSA (mg L^−1^)	Final OD_600_	rHSA OD_600_^−1^	Strain	rFDL (U L^−1^)	Final OD_600_	rFDL OD_600_^−1^
High biomass deep-well cultures^a^							
X-33 wt	13.8 ± 3.0	7.2 ± 0.2	1.9 ± 0.4	X-33 wt	nd	nd	nd
Y250 2c	0.9 ± 0.3	5.3 ± 0.2	0.2 ± 0.1	Y250 2c	nd	nd	nd
Y250 3a	4.7 ± 0.7	5.7 ± 0.3	0.9 ± 0.1	Y250 3a	nd	nd	nd
M250 1a	17.8 ± 2.0	7.3 ± 0.3	2.5 ± 0.3	M250 1a	nd	nd	nd
M250 3b	15.9 ± 2.9	8.4 ± 0.2	1.9 ± 0.3	M250 3b	nd	nd	nd

Strain	YPM rHSA^b^			Strain	BMM rHSA^b^		
	rHSA (mg L^−1^)	Final OD_600_	rHSA OD_600_^−1^		rHSA (mg L^−1^)	Final OD_600_	rHSA OD_600_^−1^
Low biomass deep-well cultures							
X-33 wt	13.8 ± 0.1	4.1 ± 0.2	3.4 ± 0.2	X-33 wt	3.9 ± 0.4	2.8 ± 0.0	1.3 ± 0.1
Y250 2c	0.4 ± 0.1	1.9 ± 0.1	0.2 ± 0.1	Y250 2c	0.7 ± 0.7	0.2 ± 0.0	3.9 ± 3.9
Y250 3a	2.0 ± 0.6	2.8 ± 0.2	0.7 ± 0.2	Y250 3a	0.1 ± 0.0	0.2 ± 0.0	0.6 ± 0.1
M250 1a	16.6 ± 2.0	5.9 ± 1.2	2.8 ± 0.2	M250 1a	7.0 ± 1.5	4.2 ± 0.2	1.6 ± 0.3
M250 3b	24.7 ± 5.7	7.4 ± 0.2	3.4 ± 0.9	M250 3b	4.7 ± 0.9	4.6 ± 0.1	1.0 ± 0.2

Strain	YPM rFDL^c^			Strain	BMM rFDL^c^		
	rFDL (U L^−1^)	Final OD_600_	rFDL OD_600_^−1^		rFDL (U L^−1^)	Final OD_600_	rFDL OD_600_^−1^
Low biomass deep-well cultures							
X-33 wt	179.4 ± 27.3	3.9 ± 0.1	38.4 ± 12.5	X-33 wt	8.5 ± 2.0	2.2 ± 0.3	3.5 ± 0.5
Y250 2c	10.3 ± 3.0	1.9 ± 0.0	5.6 ± 1.6	Y250 2c	6.0 ± 1.4	0.2 ± 0.0	36.1 ± 9.8
Y250 3a	16.9 ± 8.4	2.6 ± 0.0	6.7 ± 3.4	Y250 3a	16.4 ± 6.5	0.1 ± 0.0	122.7 ± 49.1
M250 1a	419.5 ± 26.5	3.8 ± 0,1	108.7 ± 7.5	M250 1a	65.0 ± 5.5	2.5 ± 0.1	25.6 ± 1.5
M250 3b	448.0 ± 57.3	4.1 ± 0.0	109.5 ± 13.9	M250 3b	40.0 ± 5.6	2.5 ± 0.1	17.8 ± 2.2

rHSA and rFDL were used as model proteins for expression in deep well cultures. For rHSA two screening protocols with high and low starting biomass prior to induction and growth on methanol were applied. For rFDL the low starting biomass protocol was applied. ^a^ and ^b^ number of replicates *n* = 12; ^c^ *n* = 6. Values represent averages ± standard deviation

This screening protocol allows for rather low growth rates due the high initial biomass concentration prior to switching to methanol as carbon source. Thus, in order to further analyze potential growth rate effects during recombinant protein production, we also screened the clones in YPM and BMM medium with lower starting biomass concentrations at OD_600_ = 0.2, in order to maintain cells in exponential growth for a longer time during cultivation. On YPM, the wildtype showed similar rHSA titers to the first growth protocol, despite lower final OD_600_ values. The YPM-evolved clones showed largely reduced rHSA titers and biomass yields as compared with the ancestral strain. BMM-evolved clones showed a 20 and 78% increase in rHSA levels, which correlated with increased biomass yields. A similar trend, but with overall lower rHSA yield, was observed on BMM medium (Table 4). It has to be mentioned that both YPM-evolved clones did not show any significant growth in the BMM environment, although recombinant product could be detected, resulting in a high rHSA per OD_600_ yield for clone Y250 2c.

As a second model protein, a recombinant hexosaminidase (rFDL) was expressed in order to check whether the observed trends were protein specific. Overall, the results for rFDL were very similar to rHSA expression results, with BMM-evolved clones showing significantly increased product yields on both YPM and BMM medium, although e.g. in contrast to rHSA expression, the biomass yield for the BMM-evolved clones was not higher than for the wildtype clones on YPM (Table 4).

As mentioned above, recombinant FDL expression levels are low in general and are especially low in defined minimal medium fed batch cultures (own unpublished data). Consequently, rHSA-expressing clones were used to analyze growth in bioreactor fed batch cultures. The ancestral clone and evolved clones Y250 3a, M250 1a and M250 3b clone were selected based on the results summarized in Table 4. Except for Y2503a clone, which showed low productivity, the clones from the replicates of the batch profiling were selected based on comparable rHSA yields during deep well screening. For all fed batch cultivations, an initial glycerol batch with subsequent glycerol fed batch was applied, followed by a methanol pulse for adaptation and incrementally increased constant methanol feed phases (see “Methods” section for details). Furthermore, between incremental feed rate increases, methanol pulses were applied in order to analyze substrate uptake and respiratory behavior during exponential growth. Similar to deep well batch cultures, we find varying results in terms of rHSA production (Table 5). The YPM-evolved clone Y250 3a showed lower biomass yield as well as largely reduced rHSA yields compared to the ancestral strain. The M250 1a clones showed a biomass yield similar to the ancestral strain and specific (q_P_) and volumetric productivity (Q_P_) of only about 77% of the ancestral clone. The evolved clone M250 3b showed improved recombinant protein levels in all deep well screenings. During fed batch cultivation, the biomass yield was only approximately 5% higher than the ancestral strain, but a significant increase of rHSA production with a 55 and 76% increase for q_P_ and Q_P_, was observed. Cell viability issues as potential cause for the observed differences were eliminated, since flow cytometric analysis showed high viability (>98%) throughout all fed cultivations (Additional file 1: Table S5).

**Table 5:** Results of fed batch cultivations of selected rHSA-expressing clones

Clone	YDM (g L^−1^)	rHSA (mg L^−1^)	Y_x/s_ glycerol (g g^−1^)	Y_x/s_ methanol (g g^−1^)	q_P_ (mg g^−1^ h^−1^)	Q_P_ (mg L^−1^ h^−1^)
X33 rHSA	82.2 ± 0.8	198.4 ± 0.4	0.666 ± 0.006	0.139 ± 0.004	0.009	1.39
Y250 3a rHSA	73.3 ± 0.8	34.2 ± 0.89	0.578 ± 0.011	0.093 ± 0.004	0.002	0.24
M250 1a rHSA	83.0 ± 0.6	151.7 ± 1.88	0.610 ± 0.003	0.141 ± 0.003	0.007	1.06
M250 3b rHSA	86.3 ± 0.1	350.0 ± 3.0	0.686 ± 0.011	0.149 ± 0.0	0.014	2.45

For cultivations a glycerol batch and fed batch were performed and MeOH pulses as well as constant feed phases were applied as described in the “Methods” section. YDM, rHSA yield and biomass yields (Y_x/s_) are shown. Values represent averages ± standard deviation. Specific (q_P_) and volumetric productivity (Q_P_) were calculated for the entire methanol feed process as an average for all methanol fed batch and pulse phases. Biomass yield (Y_x/s_) for glycerol was calculated for the glycerol batch phase. Biomass yield for methanol shows the average over all MeOH phases

However, differences were observed for other parameters, e.g. the length of the batch phase. For the ancestral and M250 3b strains, the glycerol batch phase was 28.9 and 29.8 h, whereas the initial glycerol batch took 35.8 and 34.3 h for clones Y250 3a and M250 1a respectively. Furthermore, a higher RQ was observed for these cultures during the glycerol batch (Table 6). The RQ values in combination with a significantly extended glycerol-batch phase are indicative of growth trade-offs on defined glycerol medium. Furthermore, increased RQ values for clones of the Y250 3a and M250 1a background during methanol pulse phases and constant feed phases were observed. Clone Y250 3a also showed a lower methanol consumption rate during the methanol pulse phases (Additional file 1: Table S6) although no *AOX* mutation was found. Thus, metabolic deficits might be linked to transcriptional rewiring caused by the *TUP1* or *ECM22* mutations found in this clone (Table 3). On the other hand, the high-producing clone M250 3b showed less pronounced differences in terms of RQ values for both glycerol and methanol phases. This clone also showed the fastest methanol consumption rate during methanol pulses (Table 6 and Additional file 1: Table S6). Besides these differences in RQ, the OTR during methanol growth was lower for methanol-adapted fed batch cultures than for the wildtype cultivation. Whereas this difference was negligible during the strongly limited constant feed phases, all three evolved clones showed OTRs ranging from 60 to 80% as compared with the wildtype fed batch culture during the MeOH pulse phases, indicating a reduced oxygen demand during exponential growth; in the case of the M250 3b clone this was despite of a faster methanol consumption rate (Additional file 1: Tables S6, S7).

**Table 6:** Respiratory quotient (RQ) of fed batch cultures during different cultivation phases

Clone	RQ glycerol batch	RQ MeOH pulse phase	RQ MeOH constant feed
X33 rHSA	0.61 ± 0.03	0.48 ± 0.02	0.52 ± 0.02
Y250 3a rHSA	0.73 ± 0.05	0.60 ± 0.03	0.62 ± 0.02
M250 1a rHSA	0.81 ± 0.08	0.58 ± 0.03	0.64 ± 0.04
M250 3b rHSA	0.59 ± 0.05	0.53 ± 0.02	0.56 ± 0.02

For MeOH phases, value of all pulse phases and constant feed phases were combined. RQ values represent average values ± standard deviation

## Discussion

### Phenotypic diversity

In order to provide an advantage in alternating environments and during niche exploration, population heterogeneity is a widely observed phenomenon among microbial populations; the basic driving force behind this heterogeneity being mechanisms such as epigenetic regulation and stochastic effects during gene regulation [52]. Thus, it is not surprising that we find differences in terms of growth rates among the individual clones that were isolated from a single population. It is interesting to observe higher growth rates of evolved populations on non-evolutionary carbon sources such as YPD (Table 1) but lower or decreased growth rates as compared to the ancestral strain for the randomly picked single clones (Additional file 1: Figure S3). It can be concluded that the single clones isolated from each population do not capture the geno- and phenotypic diversity present in a single population after 250 generations or approximately 10^10^ cumulative cell divisions. This observation is also supported by the fact that the two re-sequenced genomes from clones isolated from the same population (Y250 3a and 3c, Table 3) do not share any mutations and thus represent two independent lineages in this population.

Furthermore, YPM-evolved clones showed a significantly higher variance during growth on BMM (Fig. 1), whereas BMM-evolved clones did not display such a high degree of variance during growth on YPM medium. Thus, we conclude that long-term adaptation to growth on BMM results in the selection of phenotypes that are generally more robust and compatible with different environmental conditions as compared with methanol adaptation in rich (YP) medium. Generally our data indicate partially dual adaptation towards carbon source on the one hand and environment (nutrient rich vs. poor) on the other hand.

### Genomic mutations upon adaptive evolution

In general, DNA replication is a high fidelity process and mutations are relatively rare. However, considering the relatively large population size of microbial cultures, mutations are frequent and can be fixed within a 100–200 generation experiment (for a review see [27]). On average, we identified 2.4 mutations per clone. This number is comparable to the number observed in similar experiments in pro- and eukaryotic microorganisms. A recent study for *E. coli* showed that cultures underwent approximately 10^11.2^ total cumulative cell divisions in order to produce a new stable phenotype [31]. Furthermore, these phenotypes were based on two to eight mutations for the evolved *E. coli* populations. The results are in agreement with the high phenotypic diversity observed at a lower CCD for *P. pastoris* cultures in the current study. Our data suggest similar numbers of fixed mutations and CCDs in order to achieve a stable phenotype across different microbial species.

Regarding the large number of recent studies dealing with laboratory evolution, an intriguing question is to which extent mutations are selective, deleterious or neutral. Under neutral selection, the occurrence of synonymous mutations might be readily expected but neutral and deleterious mutations can also hitchhike under selective conditions [53]. As already observed by Lenski et al. in his large-scale experiment with *E. coli* and in *S. cerevisiae* studies, the lack or underrepresentation of synonymous mutations suggests that the majority of mutations during ALE may be selective [30, 54]. In this context, our current study did not identify any synonymous mutations in the evolved clones, suggesting that most mutations may have been selected for and correlate with the observed growth phenotypes.

### Mutational targets and functional complexes

**Fig. 3:**
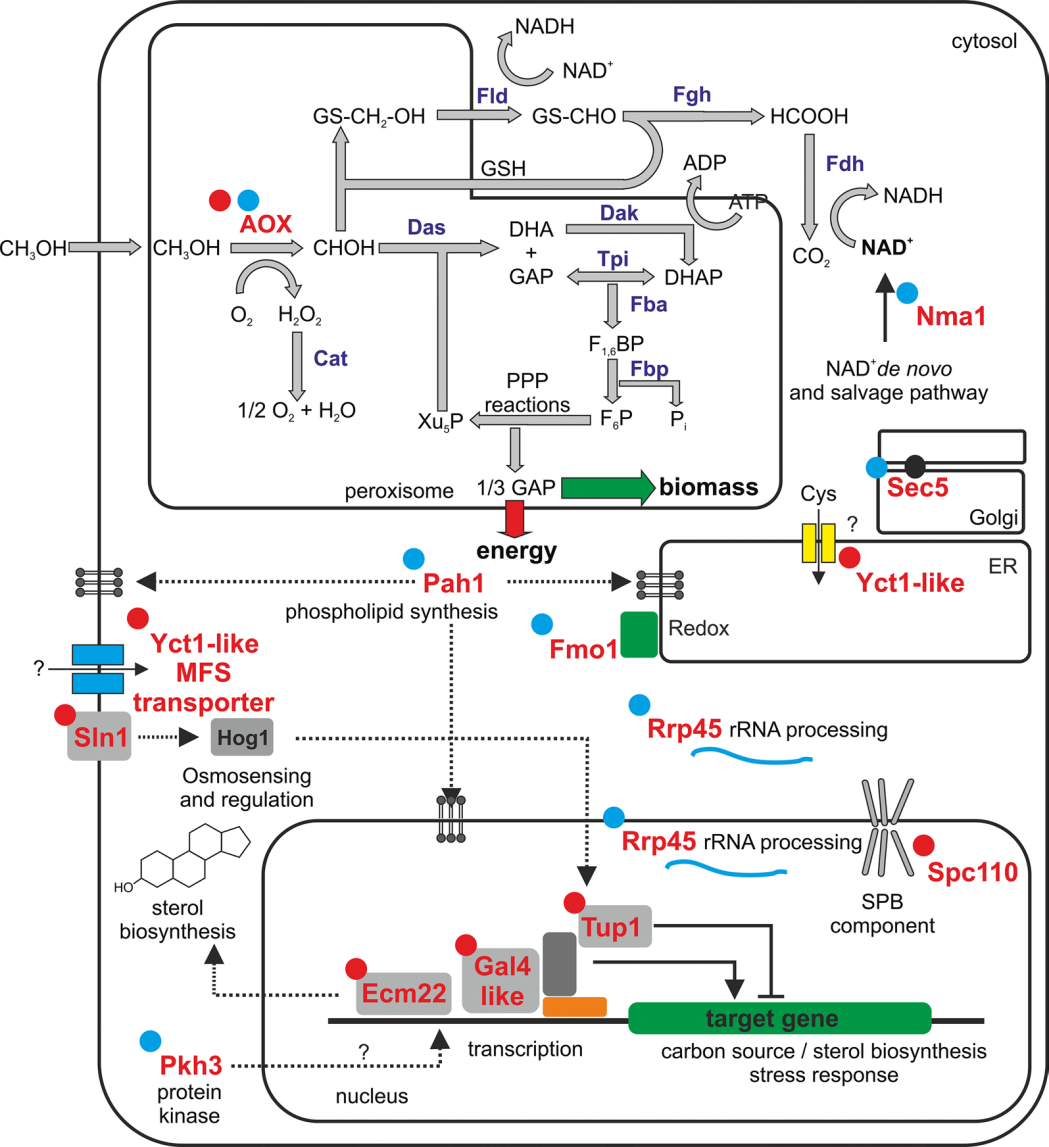
Schematic presentation of cellular pathways and genetic/protein changes. The corresponding proteins of the genes where mutations were observed (as discussed in the main text) are highlighted in *red*. *Red dots* in the* upper left* corner of the genes/proteins indicate mutations found in clones evolved on YP-medium and* blue dots* indicate mutations found in clones adapted to BM-medium. Mut-pathway: *AOX* alcohol oxidase, *Cat* catalase, *Dak* dihydroxyacetone kinase, *Das* dihydroxyacetone synthase, *Fba* fructose-1,6-bisphosphate aldolase, *Fbp* fructose-1,6-bisphosphatase, *Fld* formaldehyde dehydrogenase, *Fgh S*-formylglutathione hydrolase, *Fdh* formate dehydrogenase, *Tpi* triosephosphate isomerase

Several mutations were discovered by genome sequencing and, to a certain degree, an overlap with respect to the targets was observed (Table 3; Fig. 3). Apart from the *AOX1* locus, we did not identify convergent genes or functional complexes when comparing YPM and BMM evolved populations:

For growth on YPM, mutations in *AOX1*, *ECM22* and a novel putative transcription factor (PAS_chr2-1_0445) seem to be of particular importance as they appear in multiple independently evolved clones. In the case of *ECM22*, even the same amino acid was affected, in one case leading to a W95C, in the second case to a W95L conversion. Regarding the PAS_chr2-1_0445, we over-expressed the corresponding gene in order to analyze whether a higher gene dosage has an effect on growth. Although only minor differences were observed in comparison with the control, our data indicate that this putative transcription factor might be involved in a general environmental response and is not directly linked to methanol-adaptation. Tendencies towards decreased growth rates in YPM and YPD medium upon gene overexpression were observed (Additional file 1: Figure S4). Thus, mutations emerging in the PAS_ch2-1_0445 locus may be under pleiotropic stabilizing selection with the additional mutations identified in the respective clones. A recent random mutagenesis study identified mutations in a different *P. pastoris* GAL4-like transcriptional regulator (*ATT1*) to confer improved fitness under thermal stress and in glyco-engineered *P. pastoris* strains [55]. *S. cerevisiae* Gal4 is involved in the regulation of galactose metabolism [56], whereas the GAL4-like zinc cluster protein family is ubiquitous in fungi and serves several highly diverse functions including metabolic and stress response functions [57]. In the *P. pastoris* genome more than 20 GAL4-like proteins can be found. Considering the inability of this yeast to grow on galactose, these transcriptional regulators are likely to be involved in the regulation of other cellular pathways and may therefore represent critical selection targets in random mutagenesis and ALE approaches for improved growth.

Further mutations with probable consequences for cellular regulation were found for YPM in the *TUP1* transcriptional repressor and for BMM-evolved clones in *PKH3* and *RRP45*. Transcriptional modulation was previously exploited in *S. cerevisiae* for e.g. improved ethanol tolerance [58]; therefore, judging from multiple mutational targets related to regulatory processes, we conclude that transcriptional rewiring may represent a preferred route towards improved growth on methanol-based growth media for *P. pastoris*.

### The *AOX1* gene as convergent target

Since the *AOX1* promoter is strongly induced during growth on methanol, it is one of the most prominent promoter systems for protein production in *P. pastoris*, although issues related to the toxicity and safety of methanol have been emphasized [13]. Therefore, it is not unusual that several attempts such as promoter engineering were previously evaluated [17]. *P. pastoris* harbors two genes for the alcohol oxidase gene, *AOX1* and *AOX2.* Whereas the wildtype strain with both of these genes grows fast on methanol (Mut^+^ phenotype), impairing *AOX1* function leads to slower growth and the so-called Mut^s^ phenotype. As mentioned in the “Background” section, both cell types have been evaluated for recombinant protein production with inconclusive results. In this context, it is noteworthy that in the current study, long-term adaptation resulted in the selection of clones with mutations in the *AOX1* gene and subsequently reduced alcohol oxidase activity (Fig. 2). Since the loss of Aox1 activity generally results in a Mut^s^ growth phenotype it is surprising that three out of the four clones with *AOX1* mutations and reduced alcohol oxidase activity in this study showed increased growth rates as compared with the ancestor. We identified at least one additional mutation in each of these clones and it is very likely that these additional mutations are also adaptive and may be selective in conjunction with reduced Aox activity. For example, the evolved *P. pastoris* clone Y250 3c, with an additional mutation in the high osmolarity glycerol (HOG) pathway sensor *SLN1* [51], showed a decreased growth rate on YPM and BMM as compared with the ancestor but was still present in the population after 250 generations. This indicates that growth rate alone might not be an accurate denominator of fitness as the additional mutation might confer a selective advantage in other growth phases than exponential growth. It has been shown that the HOG pathway itself is involved in multiple cellular stress responses [59, 60]. Thus, whereas this mutation might provide a selective advantage in nutrient rich growth conditions, the modulation of HOG signaling may also be responsible for the poor growth performance in BMM.

Furthermore, previous studies already identified NADH recycling via methanol dissimilation as a major bottleneck during biotransformation in *P. pastoris* [61]. In this context, changes of both Aox activity and altered NAD synthesis rates (*NMA1* mutation) in clone M250 2c may confer an advantage during growth on minimal medium and methanol (Table 3; Fig. 3).

While Aox activity is certainly a prerequisite for growth on methanol as a carbon source, reduced Aox activity does not necessarily lead to reduced growth rates or fitness. Our data rather indicate higher-order pleiotropic effects with cellular processes such as signaling, transcriptional regulation and co-factor synthesis. Altogether, reduced Aox activity may lead to a reduction of excess intracellular formaldehyde, decreased formaldehyde-induced toxicity and an improved balance between assimilating and dissimilating methanol utilization processes.

### Putative implications of ergosterol synthesis

In the current study, we also find mutations linked to the sterol regulatory element binding protein Ecm22, which is involved in the regulation of sterol biosynthesis. Research in the model yeast *S. cerevisiae* showed that the fungal sterol, ergosterol, plays an essential role for general membrane function, affecting membrane permeability and fluidity among others [62]. Many genes in this synthesis pathway are essential, whereas cells defective in the late steps of the biosynthesis are viable but show altered membrane structures and drug susceptibility [63]. In *P. pastoris*, a recent study showed that ergosterol synthesis was heavily up-regulated on a transcriptional level under hypoxic growth conditions [64]. Hypoxic conditions conferred a positive effect on recombinant protein production using the GAP-promoter system [65]. Furthermore, it was shown that mimicking hypoxia by fluconazole treatment, thereby inhibiting ergosterol synthesis, had a positive effect on recombinant protein production [66]. Furthermore, the growth of *P. pastoris* on methanol itself relies on peroxisomal proliferation and regulatory adaptation of cellular membrane synthesis, which includes ergosterol synthesis. *ECM22* mutations only occurred in YPM-evolved clones and selection experiments were performed in potentially oxygen-limited deep-well plates. Therefore, a closer investigation of ergosterol synthesis in various methanol growth conditions along with potential implications of hypoxic growth will be have to be done in future experiments.

### Implications for recombinant protein production

Positive as well as negative effects were observed in terms of recombinant protein production in evolved *P. pastoris* clones. As shown in Tables 4 and 5, clones adapted to YPM (rich medium) showed decreased protein production performance in shake flask as well as fed batch cultivation. On the other hand, the adaptation to minimal methanol medium (BMM) generally resulted in improved recombinant protein production in comparison to the ancestral X-33 strain (Table 4) in shake flask cultures. Under methanol-limited fed batch conditions only one of the two tested clones (M250 3b) showed improved protein production, with a protein titer of approximately 180% compared to the wildtype. Moreover, our initial screening indicated that the YPM evolved clones subsequently selected for recombinant protein production retained the ability to grow on BMM medium in non-expressing conditions (Fig. 1). In contrast to these initial results, during cultivation and recombinant protein production, these clones showed almost no growth in BMM shake flask cultures. Additionally, clone Y250 2c also had an extended glycerol batch phase and very poor rHSA productivity in fed batch cultures. It has previously been noticed that recombinant protein production influences metabolic fluxes of the *P. pastoris* host cells and can lead to increased maintenance requirements [67]. Thus, an interesting question for further experiments will be, to which extend the metabolic rewiring in YPM-evolved clones in combination with the metabolic burden imposed by recombinant protein production lead to this diverging growth phenotypes of recombinant and non-recombinant evolved strains in rich and minimal growth environments. It has also been shown that the variation of environmental parameters in combination with adjusted feed strategies can lead to improved results [65]. In this study, an incrementally increased constant methanol feed protocol was applied, resulting in low growth rates (average µ during all methanol feeds ≤0.01). It will have to be addressed to which extent faster growth rates, in combination with the altered oxygen demand of methanol adapted clones, can be exploited for optimized and sustainable bioprocesses.

As discussed in the “Background” section there are several methods that can be implemented for an ALE study. In this context, it was interesting to observe that a clone selected for fast growth by serial dilution showed increased recombinant protein titers in batch as well as in methanol-limited fed batch cultivations. Future attempts may involve more elaborate selection methods such as nutrient-limited chemostats in order to improve our understanding of *P. pastoris* methanol metabolism but our data clearly indicate the suitability of relatively simple and efficient serial dilutions for the selection of clones with improved phenotypes across different cultivation methods.

## Conclusion

We provide an initial dataset on the implications of evolutionary methanol adaptation and recombinant protein production for *P. pastoris*. Our data show that selected evolved *P. pastoris* cells, especially cells adapted to a minimal growth environment, can be directly applied as recombinant host strains as we observed increased protein production in shake flask and high cell density fed batch cultures. Our dataset also indicates a complex environment—carbon source correlation where optimized phenotypes are achieved by tuning methanol metabolism, via mutations related to alcohol oxidase, in combination with the modification of transcriptional regulators. As such, we provide a basis for extended studies of *P. pastoris* cell physiology in methanol growth environments.

## Additional file

**Additional file 1.** Additional tables and figures.

**Additional file 2. Table S4. **Genome sequencing statistics

## Abbreviations

ALE: adaptive laboratory evolution
CCD: cumulative cell division
DO: dissolved oxygen
GAP: glyceraldehyde-3-phosphate
ER: endoplasmic reticulum
HOG: high osmolarity glycerol pathway
HRP: horseradish peroxidase
Indel: insertion/deletion
MeOH: methanol
OTR: oxygen transfer rate
PBS: phosphate buffered saline
RQ: respiratory quotient
SNP: single nucleotide polymorphism
WGS: whole genome sequencing
