# Supplementary material for: Implications of evolutionary engineering for growth and recombinant protein production in methanol-based growth media in the yeast Pichia pastoris
Source: Microb Cell Fact. 2017 Mar 17;16:49. doi: 10.1186/s12934-017-0661-5 (PMC5356285; doi:10.1186/s12934-017-0661-5)
Supplement: Supplementary file 1 — Additional file 1. Additional tables and figures. [file 12934_2017_661_MOESM1_ESM.pdf]

Implications of evolutionary engineering for growth and recombinant protein production in methanol-based growth media in the yeast *Pichia pastoris*

Josef W. Moser[1,2], Roland Prielhofer[2,3], Samuel M. Gerner[4], Alexandra B. Graf[2,4], Iain B.H. Wilson[1], Diethard Mattanovich[2,3] and Martin Dragosits[1*]

[1] University of Natural Resources and Life Sciences, Vienna, Department of Chemistry

[2] Austrian Centre of Industrial Biotechnology (ACIB) 1190 Vienna, Austria

[3]University of Natural Resources and Life Sciences, Vienna, Department of Biotechnology

[4] University of Applied Sciences FH-Campus Wien, Bioengineering, Vienna

josef.moser@acib.at
roland.prielhofer@boku.ac.at
samuel.gerner@fh-campuswien.ac.at
alexandra.graf@fh-campuswien.ac.at
diethard.mattanovich@boku.ac.at
iain.wilson@boku.ac.at
martin.dragosits@boku.ac.at

Additional Information

* corresponding author contact information: Martin Dragosits, University of Natural Resources and Life Sciences, Vienna, Department of Chemistry. Muthgasse 11, 1190 Vienna, Austria. Tel. +431 476547722, e-mail: martin.dragosits@boku.ac.at

Additional Tables

name	primer 5‘3‘	purpose
NGS_PAS_chr4_0821 fw	ATCATCATTATTAGCTTACTTTCA	mutation verification
NGS_PAS_chr4_0821 rv	AATCTTCTGTACTCTGAAGAGG	mutation verification
NGS_PAS_chr4_0821 ig	ACTTGGTTACTGCTCACGGTGC	mutation verification
NGS_ PAS_chr2-1_0455 fw	ATGCCTAAAGCTACAAAGAAGA	mutation verification
NGS_ PAS_chr2-1_0455 rv	GGGAACAAATTCATTAACATTGAA	mutation verification
NGS_ PAS_chr2-1_0455 ig	TCCGCTGCATTGAAATACGCAG	mutation verification
NGS_ PAS_chr3_0836 fw	ATGAGTACCGCAGCCCCAATCA	mutation verification
NGS_PAS_chr3_0836 rv	TTCTTCAACATTCCAGTAGTCA	mutation verification
NGS_PAS_chr3_1001 fw	ATGTCGTACAACAGACCATTGC	mutation verification
NGS_PAS_chr3_1001 rv	AGTCCACTTCCAGATTCTGGCT	mutation verification
NGS_PAS_chr3_0512 fw	ATGCAGTACGTAGGTAGAGCCA	mutation verification
NGS_PAS_chr3_0512 rv	GCTGTCATCGATTCTCATTTTG	mutation verification
NGS_PAS_chr3_0512 ig	AATGGTGCCATATCTTTGTTGA	mutation verification
NGS_PAS_chr3_0956 fw	ATGTCTACCAATGAACATCAAT	mutation verification
NGS_PAS_chr3_0956 rv	TTCTCTAGAGTTCACCACTTGA	mutation verification
NGS_PAS_chr2-1_0162 fw	ATGACAGAACTACTGGAGTTGC	mutation verification
NGS_PAS_chr2-1_0162 rv	TTCGGGACTCGACTCGGCGATT	mutation verification
NGS_PAS_chr2-1_0162 ig	CTTCAAACTTTTGCCCTGGAA	mutation verification
NGS_ig_PAS_chr3_0857 fw	AAATCGATGTGGCACATGTCAA	mutation verification
NGS_ig_PAS_chr3_0857 rv	CTTTATAAATGTAATTGATAAT	mutation verification
NGS_PAS_chr4_0108 fw	ATGTCAGAAGAGTCTCCAATTG	mutation verification
NGS_PAS_chr4_0108 rv	AAACTCGTCAGAGTTTGGAACA	mutation verification
NGS_36948_fw	ATGAAACAACTACTCAGAGAAG	mutation verification
NGS_36948_rv	CATCTTGAGCTTTCTTTTCTGA	mutation verification
NGS_PAS_chr1-4_0181 fw	ATGGACCCCACAAACGATCCCA	mutation verification
NGS_PAS_chr1-4_0181 rv	ATCGGCTTTGTCGCCCATAACT	mutation verification
PAS_ch2-1_0455 fw	ACAACTATTTCGAAACGAGGAATTCATGCCTAAAGCTACAAAGA	pGAPzB cloning
PAS_ch2-1_0455 rv	GTTTTTGttcTAGAAAGCTGGCGGCCGCTTAGGGAACAAATTCATTAACATTG	pGAPzB cloning

Additional Table 1. **Oligonucleotides used in the current study**; For PCR reactions to verify Illumina results and cloning of open reading frame PAS_chr2-1_0455.

population	generations per day	total generations	Total estimated CCD
X-33 Y250 1	4.32	249.4	$10^{10.01}$
X-33 Y250 2	4.31	249.0	$10^{10.02}$
X-33 Y250 3	4.31	249.4	$10^{9.96}$
X-33 Y250 4	4.32	250.3	$10^{9.99}$
X-33 M250 1	3.35	251.3	$10^{9.63}$
X-33 M250 2	3.37	252.4	$10^{9.63}$
X-33 M250 3	3.40	254.4	$10^{9.69}$
X-33 M250 4	3.34	251.2	$10^{9.60}$

Additional Table 2. **Summary of the long-term adaptation experiment**. Daily Generations were calculated based on daily duplicate OD_{600} measurements for each population yielding a total of approximately 250 generations. The cumulative number of cell divisions (CCD) was calculated as described in the material and methods section of the main manuscript.

p-values	YPM	BMM	YPD	YPDN	BMD	BMDN	YPG	BMG
**X-33**	na	na	na	na	na	na	na	na
**X-33 Y250 1**	1.68E-04	1.70E-05	1.45E-05	7.56E-01	7.37E-01	7.57E-01	2.70E-02	7.17E-03
**X-33 Y250 2**	5.62E-01	4.07E-06	4.00E-09	9.78E-01	1.22E-01	1.33E-04	2.13E-02	4.82E-01
**X-33 Y250 3**	4.24E-02	2.26E-07	5.12E-07	8.32E-01	2.30E-01	3.01E-03	9.65E-01	1.36E-02
**X-33 Y250 4**	4.71E-01	1.11E-06	7.65E-08	3.82E-01	8.56E-03	3.93E-06	1.89E-02	1.06E-01
**X-33 M250 1**	1.04E-02	1.05E-06	1.56E-05	2.21E-06	6.78E-02	2.51E-01	6.10E-01	1.05E-01
**X-33 M250 2**	1.18E-02	1.22E-06	1.25E-04	3.93E-01	2.86E-04	1.52E-01	4.61E-03	3.92E-05
**X-33 M250 3**	5.82E-02	6.11E-06	1.70E-06	1.40E-01	4.02E-04	4.22E-04	3.35E-02	6.58E-03
**X-33 M250 4**	5.30E-02	3.86E-06	7.84E-01	3.25E-01	4.98E-05	6.19E-04	1.31E-02	2.67E-02

Additional Table 3. **Growth rate Student's t-test values**. *p*-values of growth rates (as given in Table 1 of the main manuscript) compared to the ancestral strain. A paired two-sided T-test (equal variances) was performed. YPM – YP medium 1 % MeOH; BMM – buffered minimal medium 1 % MeOH; YPD – YP medium 2 % glucose; YPDN – YPD, 500mM NaCl; BMD – buffered minimal medium 2 % glucose; BMDN – BMD 250mM NaCl; YPG – YP medium 2 % glycerol; BMG – buffered minimal medium 2 % glycerol; na (not applicable).

	viability [%]						
**clone**	**M1**	**M2**	**M3**	**M4**	**M5**	**M6**	**M7**
**X33 HSA**	99.3	99.5	99.6	*nd*	99.6	99.6	99.6
**Y250 3a HSA**	99.8	98.8	99.6	*nd*	99.5	99.3	99.1
**M250 1a HSA**	99.7	99.7	99.7	*nd*	99.7	99.6	99.4
**M250 3b HSA**	99.7	99.8	99.8	*nd*	99.8	99.7	99.4

Additional Table 5. **Viability during methanol feed phase in bioreactor cultivations.** Viability (%) of *P. pastoris* bioreactor cultivations during the methanol feed (M1-M7). *nd* – not determined.

clone	h g_{MeOH}^{-1} g_{YDM}^{-1}
**X33 HSA**	0.012 +/- 0.002
**Y250 3a HSA**	0.018 +/- 0.003
**M250 1a HSA**	0.012 +/- 0.002
**M250 3b HSA**	0.010 +/- 0.003

Additional Table 6. **MeOH consumption during MeOH pulse phases.** The time (h) to consume 1 g MeOH per g biomass is shown. Values represent averages of all pulses +/- SD.

	MeOH pulse phase	MeOH constant feed
	OTR [mM h^{-1} g_{YDM}^{-1}]	OTR [mM h^{-1} g_{YDM}^{-1}]
**X33 HSA 7**	2.4 +/- 0.3	0.9 +/- 0.2
**Y2503A HSA 4**	1.4 +/-0.2	0.9 +/-0.2
**M2501a HSA 7**	1.8 +/ -0.2	0.8 +/- 0.1
**M2503b HSA 4**	1.9 +/-0.2	0.8 +/- 0.2

Additional Table 7. Oxygen transfer rates (OTR) for each of the four fed batch fermentations during the methanol pulse and constant feed phases. Values represent averages of all pulses and constant feed phases +/- standard deviation.

Additional Figures

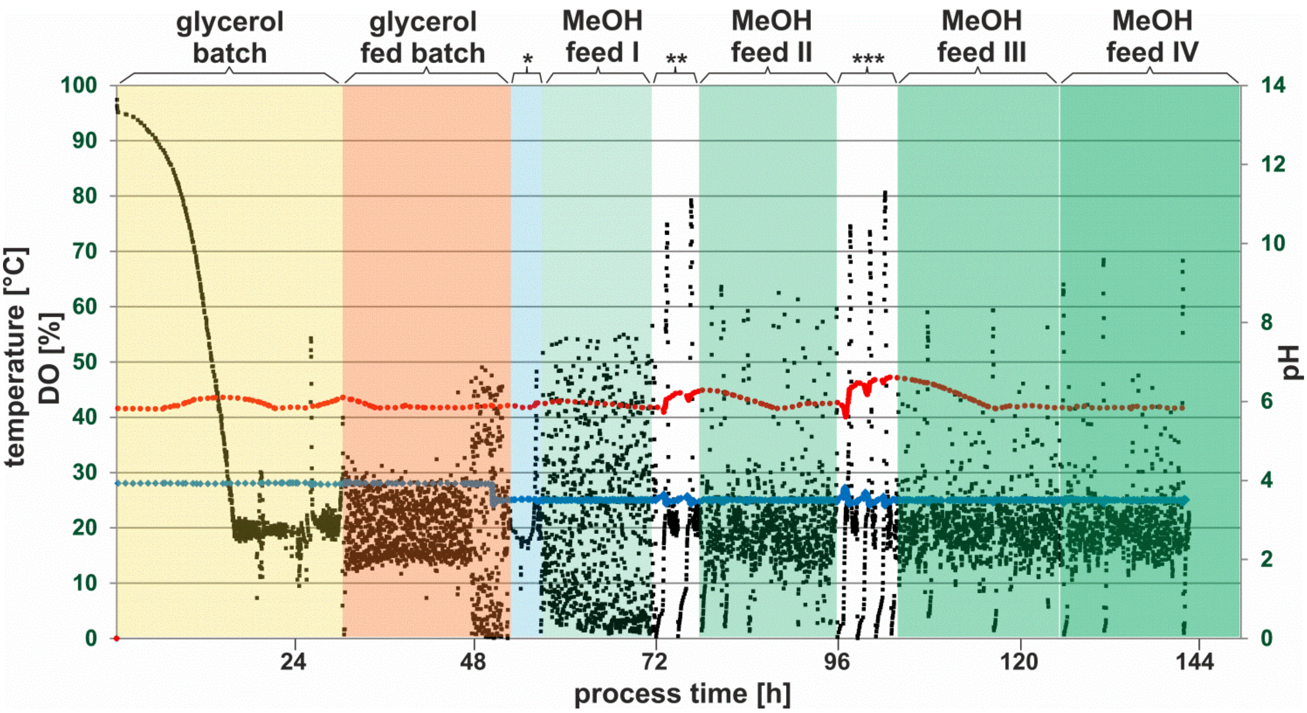

Additional Figure 1. Overview of the process setup for fed-batch fermentations. The setup of the MeOH fed batch is shown. Data for the *P. pastoris* X-33 rHSA-expressing clone were used for this exemplary plot (see main manuscript for details). Temperature (blue), dissolved oxygen (DO, black) and pH (red) are shown. Different phases of the process are highlighted. After an initial glycerol batch and fed-batch phase (2.2 – 5.0 g glycerol fed batch medium h^{-1}, depending on clone), methanol pulses and constant feed phases were applied. * MeOH adaptation pulse, 0.5 %; ** 1 and 1.25 % MeOH pulses; *** 1.5, 1.5 and 0.75 % MeOH pulses; MeOH feed I – constant feed 0.5 g h^{-1}; MeOH feed II – constant feed 1.0 g h^{-1}; MeOH feed III – constant feed 1.5 g h^{-1}; MeOH feed IV – constant feed 2.0 g h^{-1}.

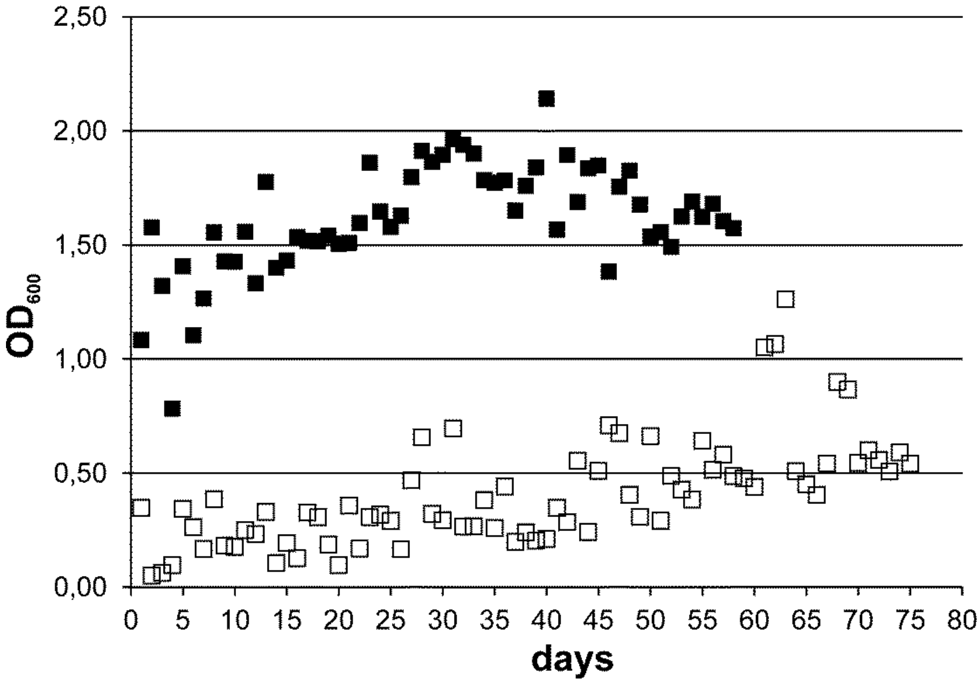

Additional Figure 2. Daily passages of *P. pastoris* X-33 populations. Four parallel populations were adapted through daily passages in YPM medium (black squares, 1:20 dilutions) and BMM medium (open squares, 1:10 dilutions used). Data points represent the average OD_{600} of all four populations.

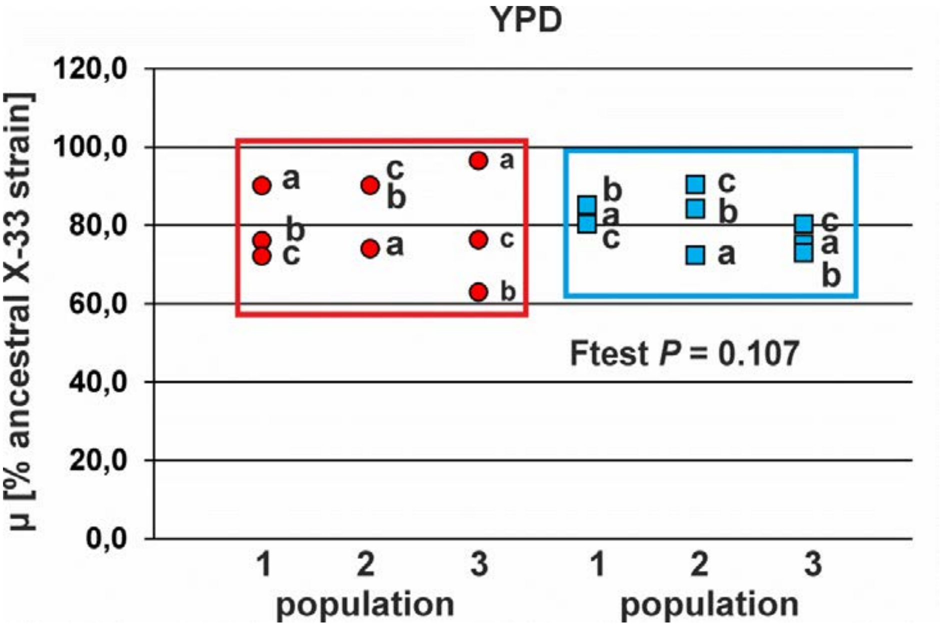

Additional Figure 3. Single clone growth rates on YPD in deep-well plates. Three single clones from YPM and BMM-evolved population 1 to 3 were randomly selected and growth rates were compared to the ancestral *P. pastoris* strain. % growth rate relative to the ancestral strain is shown. Number of replicates per single clone, $n = 2$.

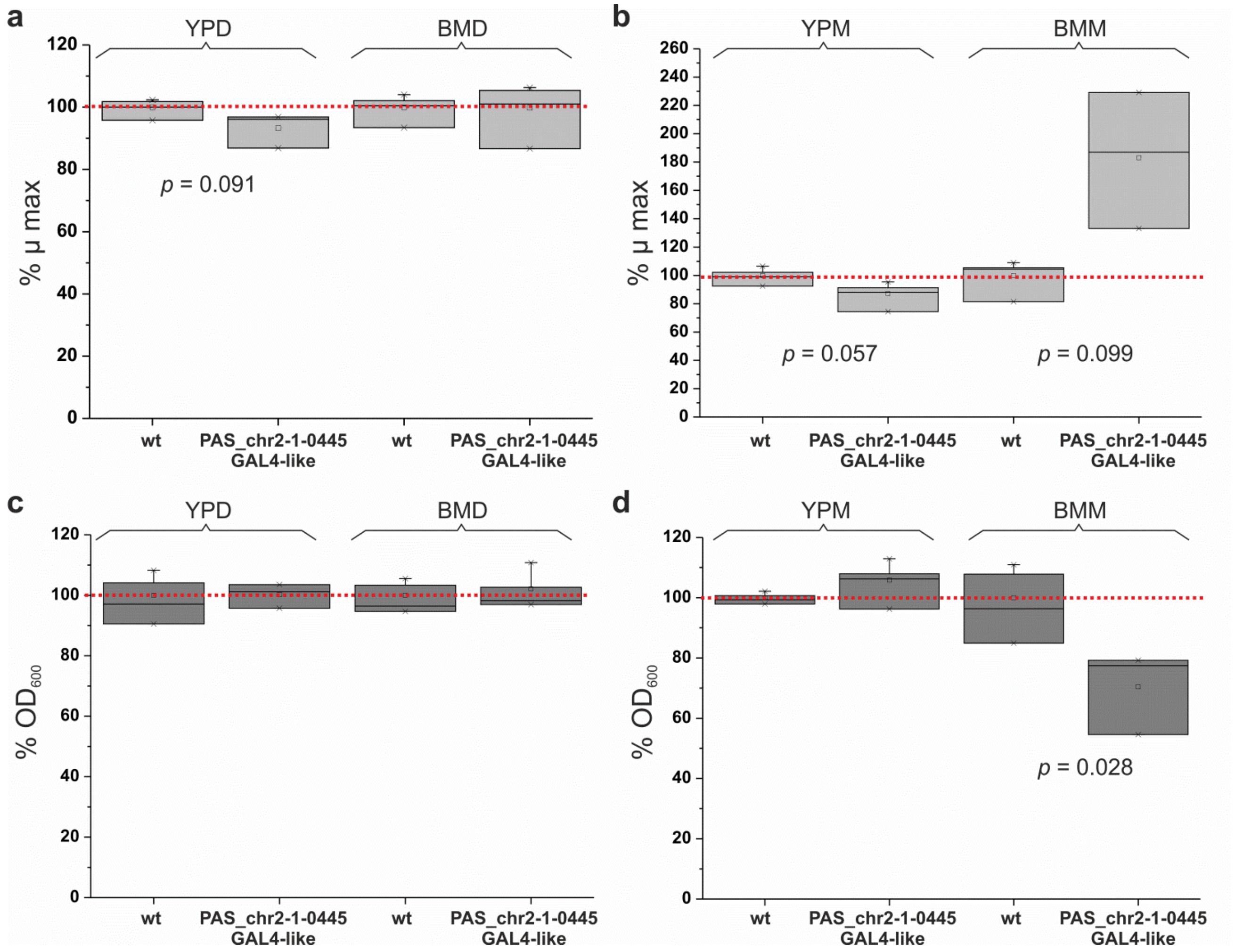

Additional Figure 4. Effect of PAS_chr2-0445 overexpression on growth. The growth of empty vector control (pGAPzB; wt) clones and PAS_chr2-0445 ORF overexpressing clones was compared in YPD and BMD medium (a,c) and YPM and BMM medium (b,d). Plots a and b show μ_{max} with the wt clones set to 100% and plots d and d show final OD_{600} after 24 hours of growth relative to the vector control. Four individual clones were randomly selected for each strain and analyzed in duplicate.

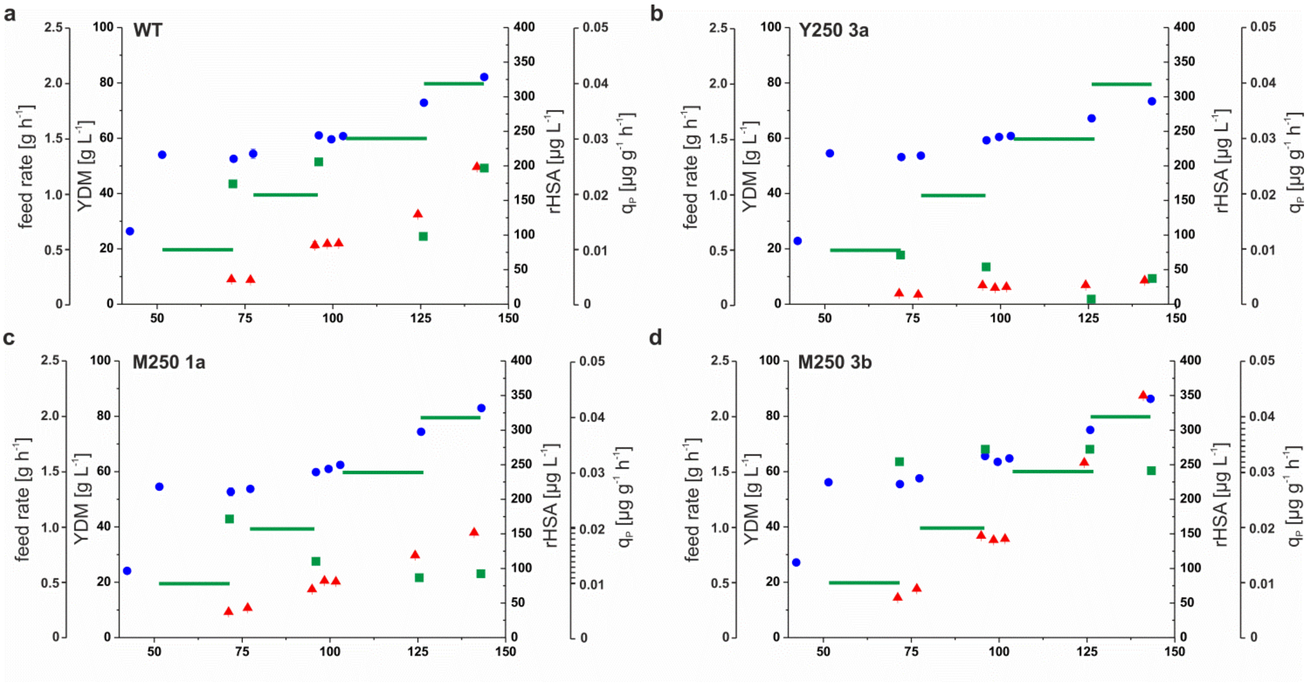

Additional Figure 5. Bioreactor cultivation profiles of rHSA-epressing X-33 (a) and methanol-adapted clones Y250 3a (b), M250 1a (c) and M250 3b (d); methanol feed rates (green lines) during constant feed phases; q_P during constant feed phases (green squares); YDM (blue dots) and rHSA titer (red triangles) are shown.

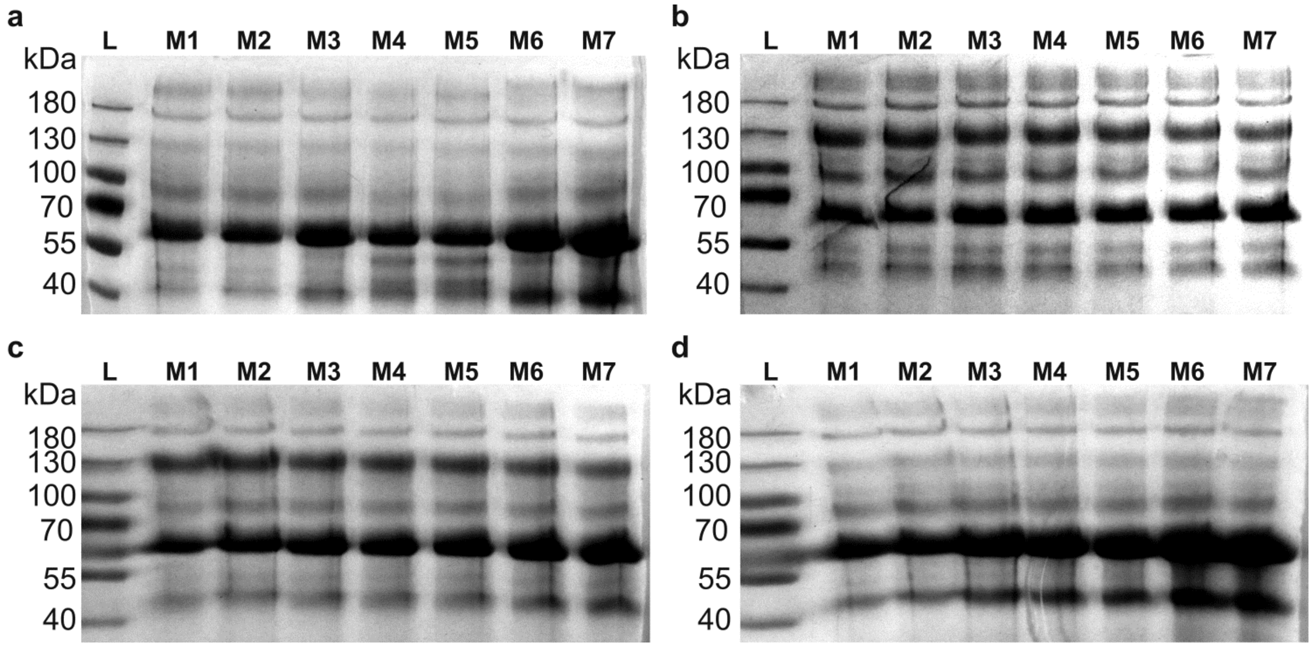

Additional Figure 6. rHSA in the culture supernatants during fed batch cultivation. rHSA production was followed throughout the methanol feed phase (samples M1 – M7). 15 µL culture supernatant were used per lane and separated on a 12 % polyacrylamide gel by SDS-PAGE, followed by collodial Coomassie G-250 staining. (A) X-33 rHSA culture (B) Y250 3a rHSA culture (C) M250 1a culture and (D) M250 3b fed batch culture
